# Factors Influencing Nutritional Status in Hospitalized Individuals Aged 70 and Above

**DOI:** 10.3390/nu16050645

**Published:** 2024-02-25

**Authors:** Raquel Ruiz-Rosso, Sara Moreno-Cámara, Belén Gutiérrez-Sánchez, Henrique da-Silva-Domingues, Rafael Del-Pino-Casado, Pedro Ángel Palomino-Moral

**Affiliations:** Department of Nursing, Faculty of Health Sciences, University of Jaén, 23071 Jaén, Spain; rrr00039@red.ujaen.es (R.R.-R.); smcamara@ujaen.es (S.M.-C.); bgutierr@ujaen.es (B.G.-S.); rdelpino@ujaen.es (R.D.-P.-C.); pamoral@ujaen.es (P.Á.P.-M.)

**Keywords:** hospitalization, older people, nutritional assessment, MNA, MNA-SF

## Abstract

Background: Older adults are vulnerable to malnutrition due to physical, psychological, and social factors. Malnutrition, a prevalent and modifiable issue in this population, is associated with an elevated risk of adverse clinical outcomes. The purpose of the study is to assess the nutritional status of older adult individuals admitted to a general hospital and examine its correlation with socio-health and demographic variables. Methods: The study included 239 individuals aged 70 and above, employing a cross-sectional descriptive observational approach with a convenience sampling method. Sociodemographic information was gathered, and variables such as cognitive impairment, functional capacity, comorbidities, medication consumption, and nutritional status were evaluated. Statistical analysis involved descriptive calculations, bivariate analysis, and multivariate analysis, utilizing binary logistic regression. Results: Approximately half of the sample were at risk of malnutrition, with a more notable prevalence among women. Factors such as age (OR = 1.04), cognitive impairment (OR = 1.06), functional dependence (OR = 0.96), and comorbidities (OR = 1.08) were linked to an elevated risk of malnutrition. In our regression model, age, cognitive impairment, and drug consumption emerged as significant predictors of malnutrition risk. Conclusions: Individuals aged 70 and above have a notably high prevalence of malnutrition risk, particularly among those experiencing functional dependence and cognitive impairment. In our sample, cognitive impairment in older adults, coupled with above-median drug consumption, emerges as the primary predictor for malnutrition risk.

## 1. Introduction

The ongoing demographic trend in Spain indicates a rapid aging of the population. Estimates for the year 2035 project that 12.8 million individuals will be aged over 65, accounting for 26.5% of the total population [1]. This progressive aging is linked to a rise in chronic and degenerative diseases, frequently correlated with malnutrition [2].

Older adults, deemed a high-risk group for malnutrition due to their physical, psychological, and social characteristics, are recommended for systematic screening as per clinical guidelines. Screening is followed by a nutritional assessment to formulate personalized support plans for those with a positive screening test [3,4].

Malnutrition stands out as a highly prevalent and modifiable issue in the older population, linked to an elevated risk of adverse clinical outcomes. These outcomes encompass frailty, osteoporosis, sarcopenia, increased morbidity and mortality in both acute and chronic conditions, and a subsequent rise in healthcare costs [5]. Various factors have been associated with the onset of malnutrition in older people. These include diminished intake resulting from acute or chronic diseases, cancer, degenerative conditions like Parkinson’s, mental health issues such as depression or dementia, and physiological changes in sensory functions. The level of dependence has also been identified as a contributing factor to malnutrition in this population [6,7,8]. Malnutrition has detrimental effects on recovery across diverse patient populations and health conditions. It impacts physiological and biochemical systems, compromises immune response, hinders muscle and respiratory function, delays wound healing, increases overall complications, prolongs rehabilitation periods, extends hospital stays, and accelerates the time to nursing home admission [2,9].

Malnutrition is a complex and multifactorial issue and, as a result, research on the risk factors for malnutrition in older people has seen continual growth in recent years [9]. The aim is to identify specific patterns and inform the design of targeted prevention strategies [10,11]. Research on potentially modifiable risk factors for malnutrition in older people is of particular interest. Notably, low physical function, including activities of daily living, strength, or performance, is supported by moderate evidence derived from consistent findings in multiple low-quality studies [9]. Marital status, however, has limited evidence, drawn from a single study of low to moderate quality [9]. Conversely, evidence on cognitive impairment or polypharmacy presents contradictory findings, with inconsistencies across studies regardless of study quality [9]. Additionally, research on non-modifiable variables such as sex or comorbidity, which require further investigation, is particularly intriguing [9,10]. These non-modifiable factors may serve as valuable risk markers.

In hospitalized individuals, the estimated prevalence of malnutrition at the time of hospital admission is 23.7% [12]. According to the EuroOOPS international study, the estimated risk of malnutrition at the time of admission for patients was 32.6% [13]. In older people, prevalence data for malnutrition vary significantly based on the environment or assessment tools used. Rates are lower in older people living in a community (7.8%) and those in functional recovery (14%). However, they are higher in nursing homes (28.4%) and hospitals (40%), and can reach up to 56% in long-stay facilities [5]. In a meta-analysis conducted by Cereda et al. [14], the prevalence of undernutrition was estimated across six healthcare settings using the Mini Nutritional Assessment (MNA) tool: community (3.1%), outpatient (6.0%), home care services (8.7%), hospital (22.0%), nursing homes (17.5%), long-term care (28.7%), and rehabilitation units (29.4%). Despite its high prevalence, malnutrition has been reported as an under-diagnosed problem in older people [15,16].

The assessment of nutritional status (NS) holds particular significance for the early detection of malnutrition, identification of risk situations, and implementation of appropriate measures. Both the Spanish Society for Parenteral and Enteral Nutrition (SENPE) (17) and the European Society for Parenteral and Enteral Nutrition (ESPEN) [2] recommend nutritional screening within 24–48 h of hospital admission using the ANM screening tool [5,17]. 

For these reasons, the objective of this study was to investigate the association between nutritional status, specifically the risk of malnutrition, and functional capacity, cognitive impairment, and comorbidity in patients aged over 70 admitted to the General Hospital of Montilla, originating from a community (home). Additionally, the aim was also to establish the relationship between nutritional status and socio-health and demographic variables.

## 2. Materials and Methods

### 2.1. Design

A cross-sectional descriptive observational study was conducted, involving 239 individuals aged over 70 who were admitted to the Hospital de Montilla (Córdoba) between July 2019 and October 2021.

### 2.2. Population and Sample

The sample was conveniently obtained through systematic consecutive recruitment of patients within 48 h of hospital admission. Inclusion criteria comprised patients aged over 70, accompanied by their family caregiver (FC). 

Exclusion criteria included patients with prior mental illnesses such as psychosis or addictions, as well as those without an FC. This study is part of a comprehensive investigation into the hospital stay of patients aged over 70 and their FCs. All information was gathered through interviews conducted in the patient’s room with the presence of the FC; additional details were extracted from the computerized clinical history.

The sample size was determined for the multiple binary logistic regression using Peduzzi’s rule [18], which recommends a minimum of 10 events per independent variable. Given a prevalence of malnutrition risk estimated at 36% [19], we aimed for 2.8 sample units (resulting in 10 events) for each independent variable. This calculation led to a requirement of 195 sample units for the seven independent variables under consideration.

### 2.3. Variables

Sociodemographic variables were gathered using a specially designed questionnaire created for this study.

#### 2.3.1. Independent Variables Included

(1) Cognitive decline: assessed using the Informant Questionnaire on Cognitive Decline in the Elderly (IQCODE) [20,21,22].
(2) Functional capacity: evaluated using the Barthel Index (BI) [23,24].
(3) Comorbidity: measured with the Cumulative Illness Rating Scale-Geriatrics (CIRS-G) [25,26].
(4) Consumption of drugs at admission (number).

#### 2.3.2. Dependent Variable

Nutritional status was assessed using the short version of the Mini Nutritional Assessment (MNA-SF) [4,27,28]. MNA-SF is a condensed form of the MNA designed specifically for screening older people. In addition to incorporating body mass index (BMI), which can be replaced by calf circumference, it includes information on relevant variables in the last three months, such as reduced food intake, weight loss, mobility, psychological stress, occurrence of acute illness, or neuropsychological problems. The MNA-SF has a sensitivity of 85%, specificity of 84%, an excellent correlation coefficient (0.90) with the long version of the MNA, and an agreement rate of 72.9%. Its predictive value has been validated by demonstrating its association with worse indicators of morbidity, mortality, and social function, as well as higher rates of physician visits. The MNA-SF screening test consists of 6 questions, categorized as follows: 11–14 points for normality, 8–10 points for risk of malnutrition, and 0–7 points for malnutrition [28,29]. In our study, we analyzed the nutritional status variable dichotomized as follows: normal nutritional status (11–14 points) and risk of malnutrition (0–10 points). In a Spanish community study, the instrument demonstrated excellent psychometric properties with a sensitivity of 85.2%, specificity of 88.9%, Positive Predictive Value of 76.4%, and Negative Predictive Value of 93.4%, along with an outstanding correlation with the total MNA [30]. The MNA is a quick and simple instrument most commonly used in older adults [31].

Statistical control variables: age (in years), sex, and marital status (categorized as married or other situations, including single, separated, or widowed).

### 2.4. Data Collection

The data were collected by three nurses who underwent prior training in the use of questionnaires and anthropometric measurements. This training included a measurement of the intraclass correlation coefficient, which was found to exceed 85%. The project was approved by the Research and Ethics Commission of the Province of Córdoba (Id: PEIBA:TD-REHPDCYCF_2018; approved in January 2019).

### 2.5. Statistical Analysis

The descriptive analysis of the main variables involved calculating means and standard deviations for quantitative variables and frequencies with percentages for categorical variables. The 95% confidence interval (CI) of the variables was also computed. To ensure the normal distribution of study variables, the Kolmogorov–Smirnov test was employed in all cases.

For both bivariate and multivariate analyses, the MNA-SF screening results were categorized into patients with normal nutrition and those at risk of malnutrition. The association with independent and control variables was examined by calculating the Odds Ratio (OR). Subsequently, a multivariate analysis was conducted using binary logistic regression, incorporating all studied independent variables (functional capacity, cognitive impairment, comorbidity, and drug consumption) and control variables (sex, age, and marital status). The adjusted Odds Ratio and its 95% confidence interval (CI) were determined.

The statistical analysis was conducted using SPSS v.22.0.0 and EpiDat 4.2. The predetermined level of statistical significance was set at 5%.

## 3. Results

Initially, 318 individuals were selected for the study. However, 41 of them (12.8%) either refused to participate, did not sign the informed consent, or were deemed ineligible due to the first exclusion criterion (mental illness or addictions, 5 cases) and the second exclusion criterion (absence of family caregiver, 17 cases). Additionally, 6 individuals died in the process, and 10 experienced early discharge or were transferred from the hospital. Our total sample comprised 239 older people, with a mean age exceeding 82 years ($\bar{x}$ = 82.21 years). The female group accounted for 58.2% of the sample, with a mean age of 83.23 years, while the male group had a mean age of 80.78 years. A significant portion, 65.7% of the participants, had no formal education. In terms of marital status, 54% were married, and 46% were widowed.

Regarding functional capacity, assessed using the Barthel Index, we found that 35.1% of the subjects were independent, 7.5% had low dependence, 19.7% had moderate dependence for some basic activities of daily living (ADL), 22.2% had severe dependence, and 15.5% were totally dependent. The mean drug consumption was 8.33, with a range from 0 to 22 (Table 1).

Regarding the main variable, nutritional status (NS), determined by screening with the MNA-SF test, we found that 50.20% (CI: 43.69; 56.72) of the sample were categorized as being at risk of malnutrition, while 49.80% (CI: 43.28; 56.30) were categorized as within normality (Table 2). Among females, 64.2% were at risk of malnutrition, compared to 35.80% of males (Table 2).

Table 2 shows the categorization of nutritional status among the independent variables included in the study. In the screening for cognitive impairment, 54.4% (CI: 47.84; 60.82) of the sample exhibited probable cognitive impairment upon admission, as indicated by the IQCODE. Among these individuals, 66.7% were found to be at risk of malnutrition. Upon categorizing the sample into dependent and independent persons using the Barthel Index, it was determined that 46.40% of the older people were functionally dependent. Within this group, 54.20% were identified as being at risk of malnutrition. Regarding comorbidity, measured by the CIRS, 42.7% of subjects exhibited higher comorbidity (more than 10 diseases), with 47.5% of them identified as being at risk of malnutrition. The risk of malnutrition was notably associated with variables such as age (over 82 years old), history of cognitive impairment, the presence of cognitive impairment as identified by IQCODE screening, and the presence of functional dependence.

Table 3 shows the Odds Ratio values for the independent variables related to the patient’s nutritional status. Age (OR = 1.04), patient’s functional status (OR = 0.96), indication of probable cognitive impairment (OR = 1.06), comorbidity (OR = 1.08), and marital status (OR = 2.15) emerged as the main variables associated with the presence of risk of malnutrition.

Finally, a binary logistic regression was conducted, encompassing all independent and control variables. The final model revealed that functional status (OR = 0.97), the state of cognitive deterioration (OR = 1.02), and drug consumption above the median (OR = 0.44) emerged as the primary predictor variables for the risk of malnutrition in the studied sample (*p* < 0.05). This model demonstrated a Nagelkerke’s R2 of 0.334 (Table 4).

## 4. Discussion

In our study, 50.20% of the sample was identified as at risk of malnutrition according to the MNA-SF test. A majority of subjects exhibited some degree of functional dependence (64.9%), probable cognitive impairment (54.4%), and comorbidities (42.7%). Age, probable cognitive impairment, functional dependence, higher comorbidity, and marital status were identified as factors associated with the risk of malnutrition. Our logistic regression model highlighted that functional status, cognitive impairment, and medication use were significant predictors of the risk of malnutrition in the studied sample.

Our study aligns with previous conclusions and makes a substantial contribution to the scientific evidence on this subject. Notably, the use of consecutive sampling without significant rejection rates minimized the risk of selection bias. The substantial sample size ensured the representativeness and reliability of our results. To enhance methodological rigor, we thoroughly controlled potential confounding variables, thereby bolstering the robustness and internal validity of our study.

The MNA-SF questionnaire has proven to be a valuable tool for identifying individuals at nutritional risk, owing to its ease of use, brevity, and lack of analytical determinations [4,5,32]. This simplicity allows for timely decision making, particularly at the time of admission. Widely adopted, the MNA is the most utilized tool for nutritional assessment of older people [31] across various social and healthcare settings, with a significant presence in hospitals. Furthermore, the recent validation of the abbreviated version, MNA-SF [29], has endorsed its use as a reliable alternative to the full MNA.

Our sample represents a geriatric population with an average age exceeding 80 years ($\bar{x}$ = 82.2 years), a majority of which were women (58.2%), aligning with the feminization trend observed in the elderly cohort [1]. An inclusion criterion in our study stipulated the simultaneous presence of the patient/family caregiver pair. Consequently, the final sample likely reflects a composition of older people from a community with a preserved family care network, omitting the representation of institutionalized individuals or those living alone. Additionally, our sample is characterized by a predominance of uneducated older individuals (65.7%), mirroring the educational landscape of older individuals in Spain. The lower educational level observed with increasing age is a characteristic of this population cohort, reflecting the historical context of their childhood.

The comparability of our data is intricately linked to several factors, including the origin and characteristics of the sample, the instrument used, and the methodology applied [31]. Notably, the MNA-SF screening categorization has recently undergone validation [29], contributing to the scarcity of data from similar studies on an international scale and within Spain. In our study, we identified that 50.20% of the participant group was at risk of malnutrition. Comparable findings have been reported in an American study utilizing the same MNA-SF screening within a day hospitalization service, revealing a rate of 45.2% of individuals at risk of malnutrition [19]. Similarly, a study of older patients admitted to a geriatric unit in Spain, employing MNA-SF screening, identified 48.2% of individuals at risk of malnutrition [33]. In a Spanish multicenter study screening institutionalized older populations with MNA-SF, values similar to ours were observed, with a risk of malnutrition of 57.9% [34]. Furthermore, our data align with another multicenter study conducted in a hospital setting in Spain, indicating similar values, with 47% of patients at risk according to the MNA [35]. 

When comparing our data with studies conducted using alternative assessment tools, notable disparities in results emerge. In the prospective multicenter EuroOOPS study, involving adults with an average age of 60 years across hospitals in 12 countries, the risk of malnutrition was estimated at 36.55% using the Nutritional Risk Screening 2002 (NRS 2002) screening tool [13]. Another study conducted in Israel on adults, utilizing the same tool, estimated that 31.5% of patients were at risk of malnutrition [36]. Two studies conducted in Spain on hospitalized patients estimated the risk of malnutrition using the NRS 2002 test at 28% [37] and 35.5%, respectively. [38]. Another Spanish outpatient study, using the Mch tool, estimated the prevalence of malnutrition in older patients at 29.6% [39].

In our study, we were unable to establish a relationship between sex and the risk of malnutrition, and other studies report similar results [40,41]. The existing literature exhibits considerable controversy regarding whether sex could play a differential role in the risk of malnutrition. In Spain, the study by Cuervo et al. [30] established an association between female sex and malnutrition in a large community sample. Additionally, other researchers have reported a similar relationship in older people residing in nursing homes [42]. In the systematic review by Chrichton et al. [43], a global association between female sex and malnutrition was established, revealing that women were 45% more likely to be malnourished than men. Further, in a regression analysis conducted on a sample of the Spanish population, it was observed that as the percentage of women in the sample increased, so did the prevalence of malnutrition [43]. The reasons explaining the association between malnutrition and sex remain unclear, likely stemming from multiple factors related to social and economic conditions, a higher probability of widowhood, gender inequality, and physiological differences [44,45]. 

Our research has revealed that being single, separated, or widowed increases the probability of malnutrition risk by 2.15 times. While the literature reviewed on the relationship between social and psychological factors has confirmed the association between marital status and malnutrition [11,46], it is noteworthy that this relationship currently lacks substantial evidence [4]. The aging process involves crucial changes related to marital status that can negatively impact nutritional status, particularly in cases of widowhood, separation, or being single. These marital statuses may be associated with a reduction in social relationships and a diminished family care network. In this context, social isolation has been identified as a factor linked to the risk of malnutrition [47].

The decline in physical function is recognized as a prognostic factor for malnutrition and mortality, significantly compromising the quality of life for older people. This decline is associated with an elevated risk of hospitalization, readmission, and institutionalization [10,11,48], with moderate evidence supporting this relationship [9]. Several explanatory factors, including pathological processes, hospital procedures, and prolonged hospital stays, directly impact the patient’s physical functionality and health, thereby increasing the risk of malnutrition [49,50]. Our study aligns with these established results and contributes to the growing scientific evidence in this domain. Notably, the use of consecutive sampling with minimal rejection rates, ensuring a reduced risk of selection bias, coupled with a sufficient sample size and control of potential confounding variables, enhances the robustness of our findings.

Cognitive impairment has been recognized as a potential indeterminate risk factor for malnutrition, with conflicting evidence [9,10,11]. However, our data substantiate the association between cognitive impairment and the risk of malnutrition. Cognitive impairment is intricately linked to neurodegenerative diseases, including Alzheimer’s disease and other dementias. It manifests through challenges in decision making, behavioral disturbances, functional dependence, and dysphagia, among other factors, all contributing to difficulties with food intake in older people [51]. The established relationship between cognitive impairment and malnutrition has been reinforced in previous reviews, including that of Tamura et al. [52], which predominantly analyzed cross-sectional studies involving samples from nursing homes or homes for older people. Additionally, this association has been affirmed in the review by Favaro-Moreira et al. [46] which incorporated longitudinal studies encompassing diverse environments, including day care centers, nursing homes, hospitals, and both rural and urban communities. Nevertheless, it is noteworthy that other studies, particularly reviews focusing on community-based samples, have not consistently confirmed this relationship. For instance, the rapid review by Luis-Pérez et al. [47] and the review by O’Keeffe et al. [10] on community-based samples did not find conclusive evidence supporting the link between cognitive impairment and malnutrition. Similarly, the review by Streicher et al. [11], a comprehensive meta-analysis of prospective studies with samples derived from the community, could not establish a definitive relationship.

To our knowledge, malnutrition shows a consistent relationship with age, likely arising as a combined consequence of comorbidity and frailty inherent to the aging process. Therefore, studies conducted in environments where older individuals with greater age and disease burden are concentrated, such as hospitals and residential or home care settings, are more likely to elucidate this relationship. Additionally, the association between cognitive impairment and nutritional risk appears to be reciprocal. Cognitive impairment may precede malnutrition, and conversely, malnutrition may precede the impairment of cognitive functions, influencing the severity and progression of cognitive decline [53,54]. Our study serves as confirmatory support, expanding the evidence on the relationship between cognitive impairment and the risk of malnutrition through a substantial sample of hospitalized subjects. The existing knowledge underscores the importance of jointly assessing both cognitive impairment and malnutrition in older people, particularly in healthcare settings with a higher burden of disease.

While polypharmacy in older people has been identified in the literature as a potential risk factor for malnutrition [9,10,11], our study does not conclusively support this relationship. On one hand, the cumulative effects of side effects from higher consumption of medications could contribute to malnutrition. On the other hand, it is possible that greater consumption of medications is associated with better socio-healthcare and ongoing health monitoring, creating a bidirectional causal relationship. The interplay of comorbidity with malnutrition, emphasized in previous studies [39,47], is not supported by our data.

### Limitations

The cross-sectional descriptive design employed in our study limits the establishment of causal relationships due to the absence of temporal follow-up. The prevalence of cognitive impairment among the older people in our sample may pose a limitation to the MNA-SF test’s use, as it incorporates subjective information. Therefore, evaluating instruments based on objective information for this population should be considered. Additionally, previous studies have highlighted the potential overestimation of malnutrition prevalence using conventional screening tools, given their low specificity arising from the subjective nature of the questions [55,56].

## 5. Conclusions

During the initial stages of hospital admission, individuals aged over 70 have a substantial prevalence of malnutrition risk, affecting half of the population, with a pronounced impact on those experiencing functional dependence and cognitive impairment.

Our findings suggest that variables such as age, patient functional status, indications of potential cognitive impairment, comorbidities, and marital status may serve as risk factors associated with the risk of malnutrition in the older population.

Furthermore, the outcomes derived from our regression model suggest that functional status, potential cognitive impairment, and above-median drug consumption in older people could be the primary variables associated with the risk of malnutrition in the analyzed sample.

## Figures and Tables

**Table 1:** Sociodemographic characteristics of the sample.

Characteristics		N	%	M	SD	95% CI
Age		239		82.21	6.825	81.34; 83.08
Sex	Female	139	58.2			
	Male	100	41.8			
Education level	No education	157	65.7			
	Basic education	76	31.8			
	Intermediate training	4	1.7			
	Higher Education	2	0.8			
Marital status	Single	14	5.9			
	Married	129	54			
	Separated	2	0.8			
	Widowed	94	39.3			
Town/City	Aguilar de la Frontera	44	18.4			
	Fernán Nuñez	42	17.6			
	La Rambla	25	10.5			
	Montilla	93	38.9			
	Montemayor	12	5			
	Montalbán	14	5.9			
	Other location	9	3.7			
Primary caregiver	Yes	239	100			
	No	0	0			
Drug consumption		239		8.33	4.19	7.796; 8.864
Functional ability	Independent	84	35.1			
	Low dependency	18	7.5			
	Moderate dependency	47	19.7			
	Severe dependency	53	22.2			
	Total dependency	37	15.5			
Cognitive impairment	No cognitive impairment	109	45.6			
	Probable cognitive impairment	130	54.4			
Comorbidity	Greater than or equal to 10 plus comorbidity	102	42.7			
	Less than 10 comorbidity	137	57.3			

CI = confidence interval; SD = standard deviation.

**Table 2:** Distribution of the main study variables according to nutritional status by MNA-SF screening.

		Total (n = 239) % (n) (CI 95%)	Nutritional Risk (n = 120) % (n)	Normal Nutritional Status (n = 119) % (n)	*p* Value
MNA-SF			50.20% (43.69; 56.72)	49.80% (43.28; 56.30)	
Age	<82	44.4 (106) (37.95; 50.89)	34.2 (41)	54.6 (65)	0.001 *
	>=82	55.6 (133) (49.10; 62.05)	65.8 (79)	45.4 (54)	
Sex	Female	58.2 (139) (51.62; 64.48)	64.2 (77)	52.1 (62)	0.059
	Male	41.8 (100) (35.51; 48.37)	35.8 (43)	47.9 (57)	
Marital status ^1^	Married	54 (129) (47.43; 60.41)	40.3 (27)	59.3 (102)	0.000 *
	Single/separated/ widowed	46 (110) (39.58; 52.56)	59.7 (40)	40.7 (70)	
Probable cognitive impairment	No cognitive impairment	45.6 (109) (39.17; 52.15)	33.3 (40)	58 (69)	0.000 *
	Probable cognitive impairment	54.4 (130) (47.84; 60.82)	66.7 (80)	42 (50)	
Functional capacity	Dependent	46.4 (111) (39.90; 52.98)	64.2 (77)	28.5 (34)	0.000 *
	Independent	53.6 (128) (47.01; 60.01)	35.8 (43)	71.4 (85)	
Comorbidity	Major_equal_10_comorbidity	42.7 (102) (36.32; 49.21)	47.5 (57)	37.8 (45)	0.131
	Lower_10_comorbidity	57.3 (137) (50.78; 63.67)	52.5 (63)	62.2 (74)	
Drug consumption	Higher consumption (More than 8 drugs)	55.23 (132) (48.68; 61.64)	58.3 (70)	52.1 (62)	0.335
	Lower consumption (Up to 8 drugs)	44.77 (107) (38.35; 51.31)	41.7 (50)	47.9 (57)	

* Variables significantly associated with nutritional status (MNA-SF). ^1^ Exposure category: single, separated, or widowed. CI = confidence interval.

**Table 3:** Unadjusted Odds Ratio for variables associated with risk of malnutrition.

Independent Variables	OR (CI 95%)	*p* Value
Functional capacity	0.969 (0.960; 0.978)	0.000
Comorbidity	1.086 (1.035; 1.138)	0.001
Probable cognitive impairment	1.062 (1.040; 1.084)	0.000
Drug consumption	0.778 (0.442; 1.370)	0.385
Age	1.045 (1.002; 1.090)	0.041
Sex	0.770 (0.431; 1.374)	0.376
Marital status *	2.159 (1.214; 3.837)	0.009

* Exposure category: single, separated, or widowed. CI = confidence interval; OR= Odds Ratio.

**Table 4:** Logistic regression analysis of variables associated with risk of malnutrition.

Dependent Variable: MNA-SF		
Independent Variables	OR Adjusted (CI 95%)	*p* Value
Functional capacity	0.975 (0.963; 0.988)	0.000
Comorbidity	1.019 (0.961; 1.080)	0.476
Probable cognitive impairment	1.028 (1.001; 1.056)	0.050
Drug consumption	0.440 (0.219; 0.887)	0.023
Age	0.994 (0.942; 1.048)	0.656
Sex	0.846 (0.418; 1.714)	0.722
Marital status *	1.347 (0.649; 2.794)	0.424
R2 of Nagelkerke = 0.334		

* Exposure category: single, separated, or widowed. CI = confidence interval; OR = Odds Ratio; MNA-SF = Mini Nutritional Assessment-Short Form.

## Data Availability

The raw data supporting the conclusions of this article will be made available by the authors on request.
